# Absence of Cardiovascular Manifestations in a Haploinsufficient Tgfbr1 Mouse Model

**DOI:** 10.1371/journal.pone.0089749

**Published:** 2014-02-24

**Authors:** Marjolijn Renard, Bram Trachet, Christophe Casteleyn, Laurence Campens, Pieter Cornillie, Bert Callewaert, Steven Deleye, Bert Vandeghinste, Paula M. van Heijningen, Harry Dietz, Filip De Vos, Jeroen Essers, Steven Staelens, Patrick Segers, Bart Loeys, Paul Coucke, Anne De Paepe, Julie De Backer

**Affiliations:** 1 Center for Medical Genetics, Ghent University Hospital, Ghent, Belgium; 2 Institute Biomedical Technology - Biofluid, Tissue and Solid Mechanics for Medical Applications, Ghent University, Ghent, Belgium; 3 Department of Morphology, Faculty of Veterinary Medicine, Ghent University, Merelbeke, Belgium; 4 Department of Veterinary Sciences, Faculty of Pharmaceutical, Biochemical and Veterinary Sciences, University of Antwerp, Wilrijk, Belgium; 5 Institute Biomedical Technology – Medical Image and Signal Processing Unit, Ghent University, Ghent, Belgium; 6 Molecular Imaging Center, University of Antwerp, Wilrijk, Belgium; 7 Departments of Cell Biology and Genetics, Radiation Oncology and Vascular Surgery, Erasmus MC, Rotterdam, The Netherlands; 8 McKusick-Nathans Institute of Genetic Medicine, Johns Hopkins University School of Medicine, Baltimore, Maryland, United States of America; 9 Howard Hughes Medical Institute, Johns Hopkins University School of Medicine, Baltimore, Maryland, United States of America; 10 Laboratory of Radiopharmacy, Faculty of Pharmaceutical Sciences, Ghent University, Ghent, Belgium; 11 Center for Medical Genetics, Antwerp University Hospital, Edegem, Belgium; The Hospital for Sick Children, Canada

## Abstract

Loeys-Dietz syndrome (LDS) is an autosomal dominant arterial aneurysm disease belonging to the spectrum of transforming growth factor β (TGFβ)-associated vasculopathies. In its most typical form it is characterized by the presence of hypertelorism, bifid uvula/cleft palate and aortic aneurysm and/or arterial tortuosity. LDS is caused by heterozygous loss of function mutations in the genes encoding TGFβ receptor 1 and 2 (*TGFBR1* and *−2*), which lead to a paradoxical increase in TGFβ signaling. To address this apparent paradox and to gain more insight into the pathophysiology of aneurysmal disease, we characterized a new *Tgfbr1* mouse model carrying a p.Y378* nonsense mutation. Study of the natural history in this model showed that homozygous mutant mice die during embryonic development due to defective vascularization. Heterozygous mutant mice aged 6 and 12 months were morphologically and (immuno)histochemically indistinguishable from wild-type mice. We show that the mutant allele is degraded by nonsense mediated mRNA decay, expected to result in haploinsufficiency of the mutant allele. Since this haploinsufficiency model does not result in cardiovascular malformations, it does not allow further study of the process of aneurysm formation. In addition to providing a comprehensive method for cardiovascular phenotyping in mice, the results of this study confirm that haploinsuffciency is not the underlying genetic mechanism in human LDS.

## Introduction

Although monogenetic disorders are rare, they offer a valuable perspective for the study of common disease processes. The Marfan syndrome (MFS), caused by mutations in the fibrillin-1 (*FBN1*) gene [1], has for example been successfully used as a model to study the complex pathophysiology of aneurysm formation in the thoracic aorta. Since *FBN1* encodes the fibrillin-1 protein, which is a major component of extracellular matrix microfibrils [2], conventional knowledge held that most manifestations in MFS, including aortic aneurysm formation, result from an inherent structural weakness of connective tissues containing abnormal microfibrils [3], [4]. The study of the pathophysiology of MFS in genetically modified mouse models recapitulating human disease, has extended this knowledge by demonstrating that fibrillin-1 also plays an important functional role in matrix sequestration of transforming growth factor beta (TGFβ), which is crucial for regulating TGFβ activation and signaling [5]. Studies in different mouse models have shown that perturbation of TGFβ sequestration contributes to the pathogenesis of the disease [5]–[8]. Subsequent studies in various tissues from patients with MFS have confirmed altered TGFβ signaling in humans [9], [10].

Based on these observations, inhibition of the TGFβ signaling pathway in mouse models by means of TGFβ inhibiting agents, such as the angiotensin II type I receptor blocker losartan, has resulted in a significant reduction in aortic root growth and rescue of aortic wall architecture [7]. Additional and very convincing evidence for involvement of the TGFβ pathway in aneurysmal disease was provided by the identification of *TGFBR1* and *−2* mutations in patients presenting a phenotype characterized by aortic root aneurysm, arterial tortuosity and craniofacial malformations (including hypertelorism and bifid uvula/cleft palate), called the Loeys-Dietz syndrome (LDS) [11]. Aortic aneurysms in LDS tend to evolve more aggressively than in the MFS with rapid growth and early dissection in most (but not all) cases [12]. Mutations in LDS most commonly reside within the intracellular serine/threonine kinase domain of either the *TGFBR1* (1/3 of patients) or *TGFBR2* (2/3 of patients) gene, encoding the TGFβ receptors [13]. The vast majority of all mutations identified in *TGFBR1* and *TGFBR2* are missense mutations, however, other mutation types, including nonsense (*TGFBR1* and *TGFBR2*), frameshift (*TGFBR2*) and splice site (*TGFBR1* and *TGFBR2*) mutations have also been identified [12], [14] (Table S1). So far, no significant genotype-phenotype correlations have been delineated when comparing both genes or different types of mutations. Previous co-transfection experiments suggested a mild dominant-negative effect for *TGFBR1* and *−2* missense mutations resulting in loss of signaling potential of the receptors [15], [16]. No detailed studies have been performed to investigate the effect of other TGFβ receptor gene mutation types. In aortic tissue from patients with either *TGFBR1* or *−2* mutations, unexpected upregulation of TGFβ signaling was demonstrated by an increased expression of the downstream effector, i.e. connective tissue growth factor (CTGF), and accumulation of nuclear pSmad2 [11]. This phenomenon has been referred to as the TGFβ paradox. In order to study this apparent paradox, we generated a new *Tgfbr1* mouse model aiming to mimic human LDS.

Several *Tgfbr1* and *Tgfbr2* mouse models have already been developed and studied. These models showed that both receptors are required for correct TGFβ signaling since homozygous *Tgfbr1* and *−2* knock-out mice and conditional *Tgfbr1* and *−2* knock-out models for vascular smooth muscle cells and endothelial cells die prematurely due to abnormal vasculo- and angiogenesis [17]–[20]. Interestingly, conditional deletion of *Tgfbr1* or *−2* in neural crest cells results in immediate postnatal lethality due to either cardiovascular or pharyngeal defects, including persistent truncus arteriosus, interrupted aortic arch and inappropriate remodeling of pharyngeal arch arteries [21], [22]. Although these early models for either of the TGFβ receptors clearly indicate that TGFβ is involved in various steps of cardiovascular development, they were not suitable for the study of the molecular and pathogenetic mechanism of LDS. First, all homozygous mutant mice presenting cardiovascular features die prematurely, either during embryonic development or in the early postnatal period. Second, heterozygous knock-out mice do not develop any phenotypic abnormality. These previous reports, however, all focused on the embryonic and early postnatal phase and no in depth analysis of the cardiovascular system was performed in adult heterozygous mice. This leaves the possibility that these heterozygous knock-out mice develop a cardiovascular phenotype later in life. For example, it was reported previously that heterozygous *Tgfb2* knock-out mice do not show a phenotype in contrast to the homozygous mutant mice that show late embryonic lethality secondary to congenital heart disease [23]. Following the identification of loss of function mutations in the *Tgfb2* gene in humans with thoracic aortic aneurysm, however, the mouse model was revisited and Lindsay and colleagues showed that the haploinsufficient *Tgfb2* mice (*Tgfb2+/−*) showed significant dilatation of the aortic annulus and root at the age of 8 months [24]. This observation further warrants a more in depth cardiovascular evaluation of *Tgfbr* mouse models. Theoretically, mice are good models for the study of the TGFβ receptor type 1, since the sequence homology between humans and mice is very high (91.12% genomic level, 97.19% protein level).

In a collaborative research effort between our group and the group of H. Dietz (Baltimore) several *Tgfbr1* and *−2* mouse models with either a missense or nonsense mutation in one of the receptor genes were investigated. In addition to the search for a suitable model for the study of aneurysm formation in LDS and the TGFβ paradox, this study was also set up to optimize cardiovascular phenotyping in mouse models.

## Methods

### Ethics statement

For all procedures the Principles of Laboratory Animal Care (NIH publication 86–23, revised 1985) were followed. All procedures were approved by the Ghent University Hospital ethical committee for laboratory animal testing (Permit Number: ECD07/20). All *in vivo* non-invasive imaging was performed under anesthesia, and all efforts were made to minimize suffering.

### Mice

Mice were generated by Ingenium according to the procedure described by Augustin *et al* [25]. Upon screening of a library containing mutant mouse sperm that were generated by N-ethyl-N-nitrosurea (ENU) mutagenesis in healthy C3HeB/FeJ males, a nonsense mutation was identified in exon 7 of the *Tgfbr1* gene (p.Y378*). Subsequently, these mice were backcrossed to a C57BL/6 background.

### Genotyping mice

Mice were toe-clipped and tail-clipped between postnatal day 8 and 10. The DNA fragment containing the p.Y378* mutation was amplified from crude tail lysate using the KAPA 2G^TM^ Robust Hotstart kit (Kapabiosystems). Subsequently, PCR products were sequenced using the Sanger sequencing technique on an ABI 3730XL Sequencer (Life Sciences).

### Determination of the lethal phase of homozygous mutant embryos

Pregnant female mice were euthanized by means of cervical dislocation at day 8.5–12.5 post coitus (dpc). The uteruses of the mice were dissected and embryos were collected. Embryos were either snap-frozen in liquid nitrogen for RNA isolation or fixated in Bouin solution (Sigma-Aldrich) for 2 hours and then incubated in 70% ethanol and embedded in paraffin for histological examination.

### In vivo imaging

#### Echocardiography

Prior to the imaging studies, mice were anesthetized (1.0% to 1.5% isoflurane mixed with 0.5 L/min 100% O_2_) and coat hairs were removed with hair removal cream. Anesthetized mice were placed in dorsal recumbency on a warmed pad, keeping the body temperature around 37°C. Throughout the examination the heart rate, respiration rate and body temperature were monitored. Ultrasound data of the thoracic aorta and left ventricle were obtained in 4 wild-type and 4 heterozygous mutant mice at 6 months and 9 wild-type and 9 heterozygous mice at 12 months of age with an ultrasound apparatus (Vevo 2100, VisualSonics) equipped with a high-frequency linear array transducer (MS 550D, frequency 22–55 MHz). The diameter of the aorta was measured from the parasternal, and suprasternal windows at the level of the annulus, sinus of Valsalva, ascending aorta, aortic arch, and descending thoracic aorta (Figure S1). Diameters were measured in diastole from inner to inner edge. Left ventricular dimensions were obtained in M-mode from parasternal short axis view, according to standard methods. Fractional shortening was used as parameter for left ventricular function (FS  =  (LVEDD-LVESD/LVEDD)*100; with LVEDD  =  left ventricular end diastolic diameter, LVESD  =  left ventricular end systolic diameter). Standard LV diastolic function parameters were obtained with the combination of transmitral pulsed Doppler and mitral annular TDI. Transmitral Doppler signals were obtained by placing the sample volume of the pulsed Doppler between the tips of the mitral leaflets in the apical four-chamber (4C) view. Early (E) and late (A) transmitral flow velocities, the ratio of early to late peak velocities (E/A) and deceleration time of E velocity (DT) were obtained. TDI derived indices, early (Em) and late (Am) mitral annulus velocities were recorded using pulsed wave TDI mode by positioning the Doppler cursor at the septal atrioventricular margins of the LV in the apical four-chamber images.

#### Micro-CT

Four animals of each genotype group and each time point were anesthetized with 1.5% isoflurane mixed with 0.5 L/min 100% O_2_ and, once anesthetized, Aurovist (Nanoprobes) at a dose of 150 microliter/25 gram body weight was injected intravenously in the lateral tail vein. Immediately after injection, when the contrast was maximal, the animals were scanned in supine position in a FLEX Triumph-II CT scanner (Gamma Medica-Ideas). The acquisition parameters were the following: 50 µm focal spot, 2×2 detector binning, 1024 projections over 360°, 3 times magnification, and 70 kVp tube voltage. The data was reconstructed with proprietary software (Cobra EXXIM) using a Feldkamp-type algorithm with Parker's weighing function in a 512×512×512 matrix with a 75 µm voxel size. Reconstructed images were converted into DICOM standard format, and imported into the 3D segmentation software package Mimics (Materialise). The aorta was semi-automatically segmented to select the (contrast-enhanced) lumen, requiring manual intervention to separate aortic and venous segments. The resulting mask was then wrapped and smoothed while care was being taken not to cause any shrinkage. This resulted in a 3-D reconstruction of the thoracic aorta, including the aortic arch and its three major branches (brachiocephalic artery, left common carotid artery, and left subclavian artery). Due to movement artifacts caused by the proximity of the heart, no reliable reconstruction could be made of the aortic annulus and sinus. A centerline was calculated in Mimics, and at the ascending aorta, aortic arch and descending aorta the local cross-sectional area was measured orthogonal to the centerline.

#### PET

PET imaging was conducted using the same FLEX Triumph-II (Gamma Medica-Ideas) system as used with micro-CT imaging. This system consists of a micro-PET module (LabPET8) with 2×2×10 mm^3^ LYSO/LGSO scintillators in an 8-pixel, quad-APD detector module arrangement. This allows for a 1.5 mm spatial resolution in rodents at a sensitivity of 4%, thereby covering a field-of-view of 10 cm transaxially and 8 cm axially. Both the CT and PET modules are attached to the same system, leading to perfect co-registration of both modalities.

Six mice (4 heterozygous, 2 wild-type) were fasted overnight, after which they received an intravenous injection of 19.89±1.44 MBq of [18F]-fluorodeoxyglucose (FDG). After 40 minutes of uptake without anaesthesia, the animals were anesthetized with 1.5% isoflurane mixed with 0.5 L/min 100% O_2_ and scanned for 30 minutes in two bed positions. The PET data were iteratively reconstructed by 60 iterations of the 2D MLEM algorithm with a span of 31 to obtain a 92×92×95 matrix of 0.5×0.5×1.175 mm voxels. The PET data was evaluated in VIVID (GMI) by calculating the percentage of injected dose (%ID) inside the aorta. The relevant aorta volume was determined from the aorta segmentation obtained from the contrast-enhanced micro-CT scan.

### Ex vivo fluorescence reflectance imaging

Increased activity of MMPs (matrix metalloproteinases) can be assessed using long-circulating protease-activatable near infrared fluorescent probes. These autoquenched fluorescence probes convert from a non-fluorescent to a fluorescent state by proteolytic activation and can be used as a sensitive readout that reflects subtle changes in protease activity in the extracellular matrix in pre-aneurysmal lesions [26]. The proteolytic activity comes from MMPs that can cleave an MMP-specific recognition sequence between the carrier and the fluorochromes of these probes [27]. Four mice (2 heterozygous T*gfbr1* mice, 2 wild-type mice) were injected via tail vein injection with 5nmol MMPsense 680 (Perkin Elmer). Twenty-four hours after injection the animals were sacrificed and aortas were harvested for *ex vivo* examination using the Odyssey imaging system. Near infrared images were obtained at the 700 nm channels and analysed on relative fluorescence.

### Vascular corrosion casting

The vascular corrosion casting procedure was performed as previously described [28]. In short, mice (8 wild-type and 8 heterozygous mutant mice of both 6 and 12 months old) were starved 24 hours before sacrificing them by means of CO_2_ asphyxiation. The abdominal aorta was dissected and 3 ml of Batson polymer (Polysciences) was injected through a 26 gauche catheter. After polymerization, mouse bodies were macerated overnight in 25% potassium hydroxide and rinsed. The casts of the arterial blood vessels were evaluated using a dissecting microscope with 5-megapixel camera (Leica).

### Statistical analyses

Results are presented as mean (standard deviation (SD) in parentheses). Data were analyzed with the unpaired sample t-test for normal-distributed continuous variables; non-normal distributed values were compared using the Mann-Whitney-U test. χ^2^ test was used to compare categorical variables. If not all cells had an expected count of 5 or more, Fisher's Exact test was applied. A p-value of <0.05 was used to define statistical significance (two-sided). SPSS version 20.0 was used for the statistical analysis (SPSS Inc, Chicago, IL, USA).

### Cell cultures

In order to obtain aortic smooth muscle cells, 6 wild-type and 6 heterozygous mutant mice of both 6 and 12 months old, were euthanized with CO_2_ and the thoracic aorta was dissected. The following steps were previously described by Ray and colleagues [29].

Aortic smooth muscle cells were grown in SmBM smooth muscle cell basal medium supplemented with 5% fetal bovine serum, antibiotics and antimycotics, and growth supplements (0,1% hEGF, 0,1% insulin, 0,2% hFGF-B and 0,1% GA-1000) (Lonza) at 37°C and 5% CO_2_. Cells were harvested when a monolayer was formed and RNA was isolated.

### cDNA analysis

RNA was isolated from cultured aortic smooth muscle cells from adult mice on the one hand and whole snap-frozen embryos on the other hand using the RNeasy Mini kit (Qiagen). Subsequently, cDNA was synthesized using the Superscript II reverse transcriptase kit with random hexamer primers (Invitrogen). cDNA was amplified using primers spanning the entire exon 7 of the *Tgfbr1* gene. The PCR product was analyzed on a Labchip analyzer (Calliper) and subsequently Sanger sequenced on an 3730XL Sequencer (Life Sciences).

### Quantitative real-time PCR

RT-qPCR was carried out on cDNA samples using the Roche 5× mastermix and resolight dye (Roche) on the LC480 machine (Roche). All reactions were carried out in duplicate and normalized to the geometric mean of two reference mouse specific repeat sequences.

### Western blot analysis

Proteins were isolated from snap-frozen thoracic aortic tissue of 2 heterozygous and 2 wild-type mice for each time point. The lysis buffer (RIPA, Sigma-Aldrich) was complemented with protease (Roche) and phosphatase inhibitors (Sigma-Aldrich). Protein samples were reduced by boiling and adding dithiothreitol (DTT) and loaded on a NuPage 4–12% Bis-Tris gel (Invitrogen) together with a 5× non reducing lane marker sample buffer (Thermo Scientific). Following SDS-PAGE, the proteins were transferred onto a nitrocellulose membrane using the iBlot dry blotting system (Invitrogen). The membranes were blocked in 2% ECL advantage blocking buffer (GE Healthcare) and incubated overnight at 4°C with primary antibody directed against phosphorylated p44/42 MAPK (ERK1/2 XP^TM^ rabbit mAb (Cell Signaling Technologies) (1/1000)). Subsequently, the membranes were incubated with secondary anti-rabbit IgG HRP-linked antibody (Cell Signaling Technologies) (1/5000). Membranes were developed with the SuperSignal West Dura chemiluminescent substrate (Pierce). Membranes were then stripped and re-blocked, in order to incubate with a primary antibody directed against the non-phosphorylated form of p44/42 MAPK (ERK1/2) (Cell Signaling Technologies). Next, the membranes were again incubated with the secondary antibody and developed. Quantification of the signal was performed using Image J software (NIH).

### Histological, immunohistochemical and immunofluorescent analyses

Paraffin-embedded thoracic aortic tissue samples from 4 heterozygous mutant and 4 wild-type mice from each time point were made. From these formalin (for embryos Bouin)-fixed, paraffin-embedded specimens, 5 *μ*m-thick sections were made. Hematoxylin-eosin and Verhoeff-Von Giesson stainings were performed according to standard protocols. For immunohistochemistry, epitopes were unmasked using 1mM EDTA pH 8 buffer (not for actin) and auto-peroxidase activity was inhibited by incubation in 3% H_2_O_2_. Sections were blocked with 5% bovine serum albumin (Sigma-Aldrich). Antibodies directed against CTGF (Abcam), pSmad2 (Ser465/467) (Cell Signaling Technologies) and smooth muscle α-actin (Sigma-Aldrich) were used. Subsequently, sections were incubated with a secondary antibody, either biotinylated goat anti-rabbit IgG (pSmad2 and CTGF) (Vectastain) or Cy3-labeled donkey anti-goat (smooth muscle α-actin) (GE Healthcare). When the biotinylated secondary antibody was used, sections were incubated subsequently with ABC (Avidin: Biotinylated enzyme Complex) reagent (Vectastain) and DAB (3,3′-Diaminobenzidine) peroxidase (Vectastain). Sections were dehydrated in xylene and mounted with Entellan (Sigma Aldrich). When the Cy3-labeled antibody was used, sections were immediately mounted with aqueous mounting medium (Vectastain).

## Results

### Development of the *Tgfbr1* mouse model

A chemical ENU mutagenesis process was used to develop *Tgfbr1* mutant mouse sperm (Ingenium). Four out of six induced mutations included an intronic mutation, two silent mutations and one missense mutation predicted to have a low pathological risk according to the small physicochemical difference between the substituted and substituting residues. One nonsense mutation and one missense mutation were of potential pathogenic interest. In this study, the mouse sperm with nonsense mutation in exon 7 of the *Tgfbr1* gene (p.Y378*) was used for *in vitro* fertilization, creating a mouse line with a germ line mutation in the *Tgfbr1* gene.

### Natural history of the *Tgfbr1* mouse model

Upon genotyping of the offspring of two heterozygous p.Y378* mice 8 days after birth, no homozygous p.Y378* mice were identified, which suggested that these mice die during embryonic development. In contrast, heterozygous mutant mice developed normally, were fertile and had a normal lifespan, similar to their wild-type littermates.

**Figure 1:**
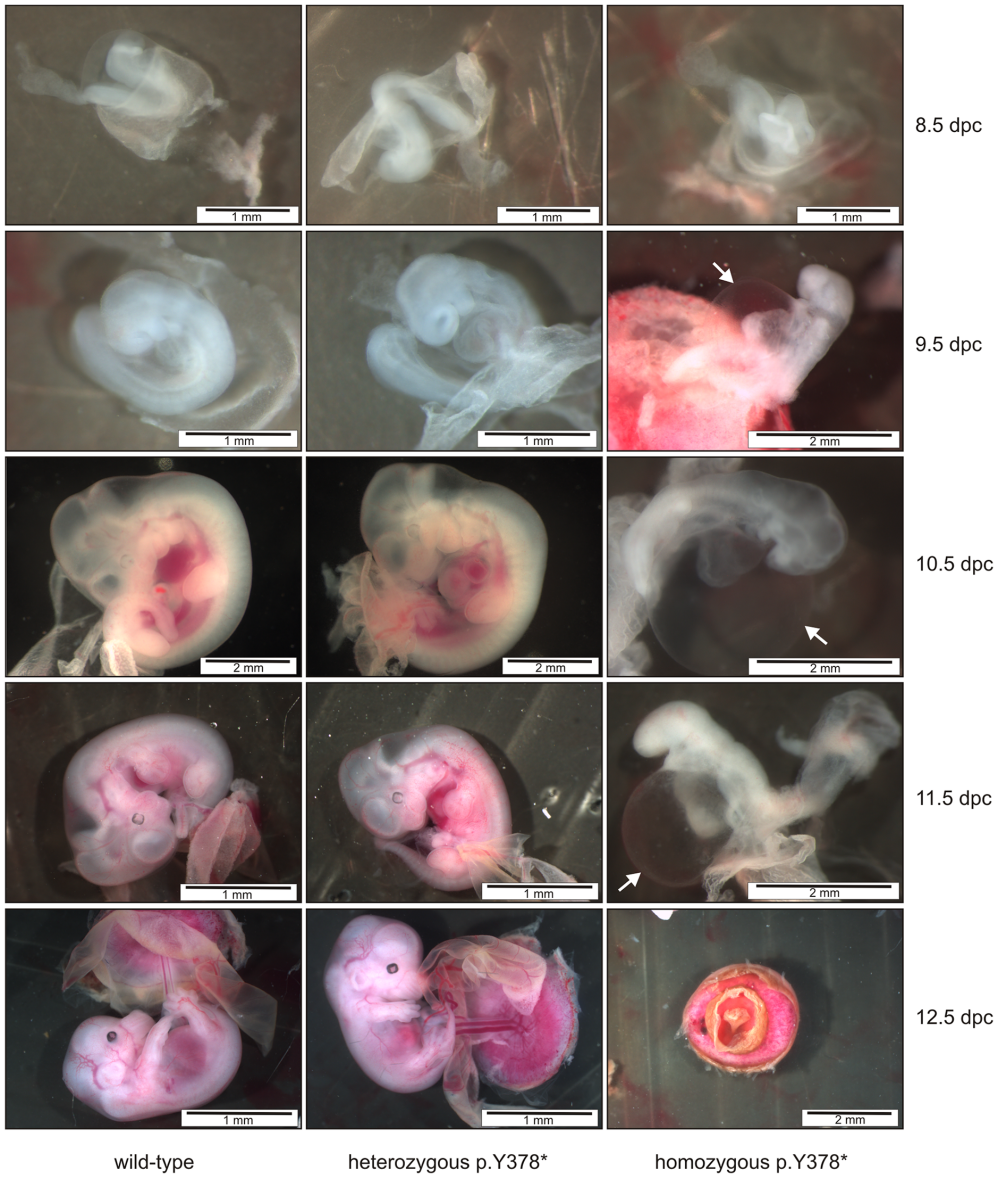
Morphology of wild-type, heterozygous and homozygous mutant mouse embryos at different time points. At 8.5 dpc mutant embryos are indistinguishable from their wild-type littermates. From 9.5 dpc onwards homozygous mutants show an abnormal development characterized by growth retardation and enlarged pericard (white arrow, right panel). By embryonic day 11.5 most of the embryos died and complete resorption is a fact by day 12.5. Heterozygous mutant *Tgfbr1* embryos and wild-type embryos develop normally.

To determine the lethal phase of the homozygous mutant mice, pregnant females were sacrificed at 8.5 through 12.5 days post coitus (dpc). This time frame was selected based on the lethal phase of homozygous *Tgfbr1* and *−2* knock-out mice [19], [20]. At 8.5 dpc the homozygous mutant mice were indistinguishable from their heterozygous mutant and wild-type littermates. By day 9.5, homozygous mutant mice showed developmental delay, enlarged pericardium, and defective vascularization of the yolk sac. By day 11.5 all homozygous mutant embryos died. Complete resorption occurred by embryonic day 12.5 (Figure 1).

### The cardiovascular system

**Figure 2:**
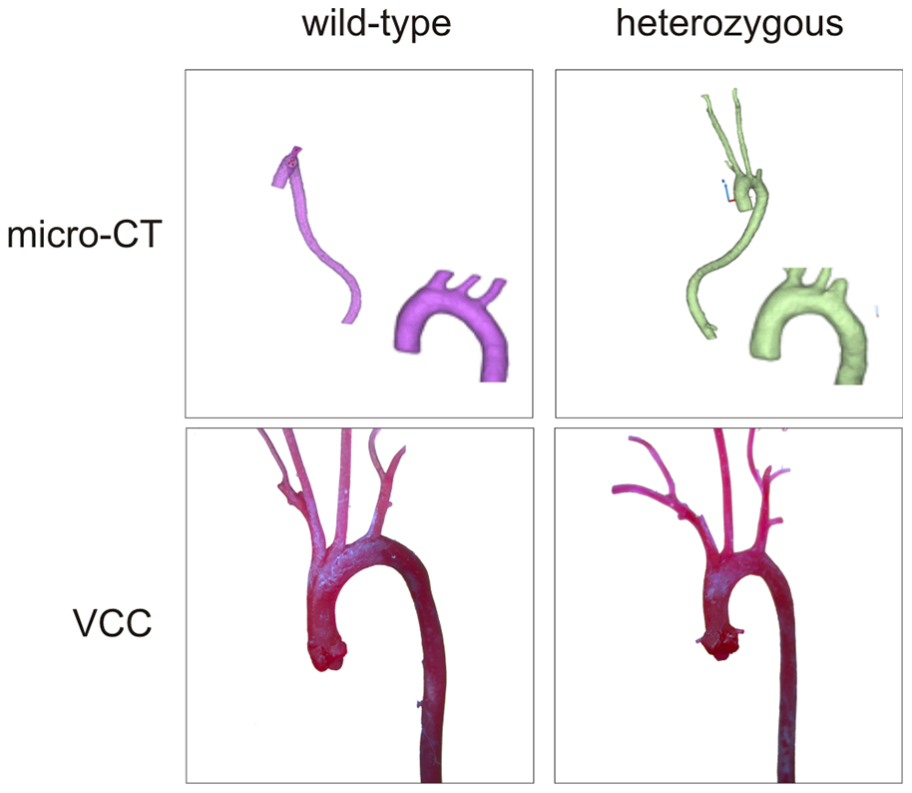
3-D reconstruction micro-CT images and vascular corrosion casts of wild-type and heterozygous *Tgfbr1* mice. No aortic aneurysm nor aortic/arterial tortuosity was observed by micro-CT (upper panel) or vascular corrosion casting (VCC – lower panel) in heterozygous mice (right) compared to wild-type controls (left) (6 months old).

**Figure 3:**
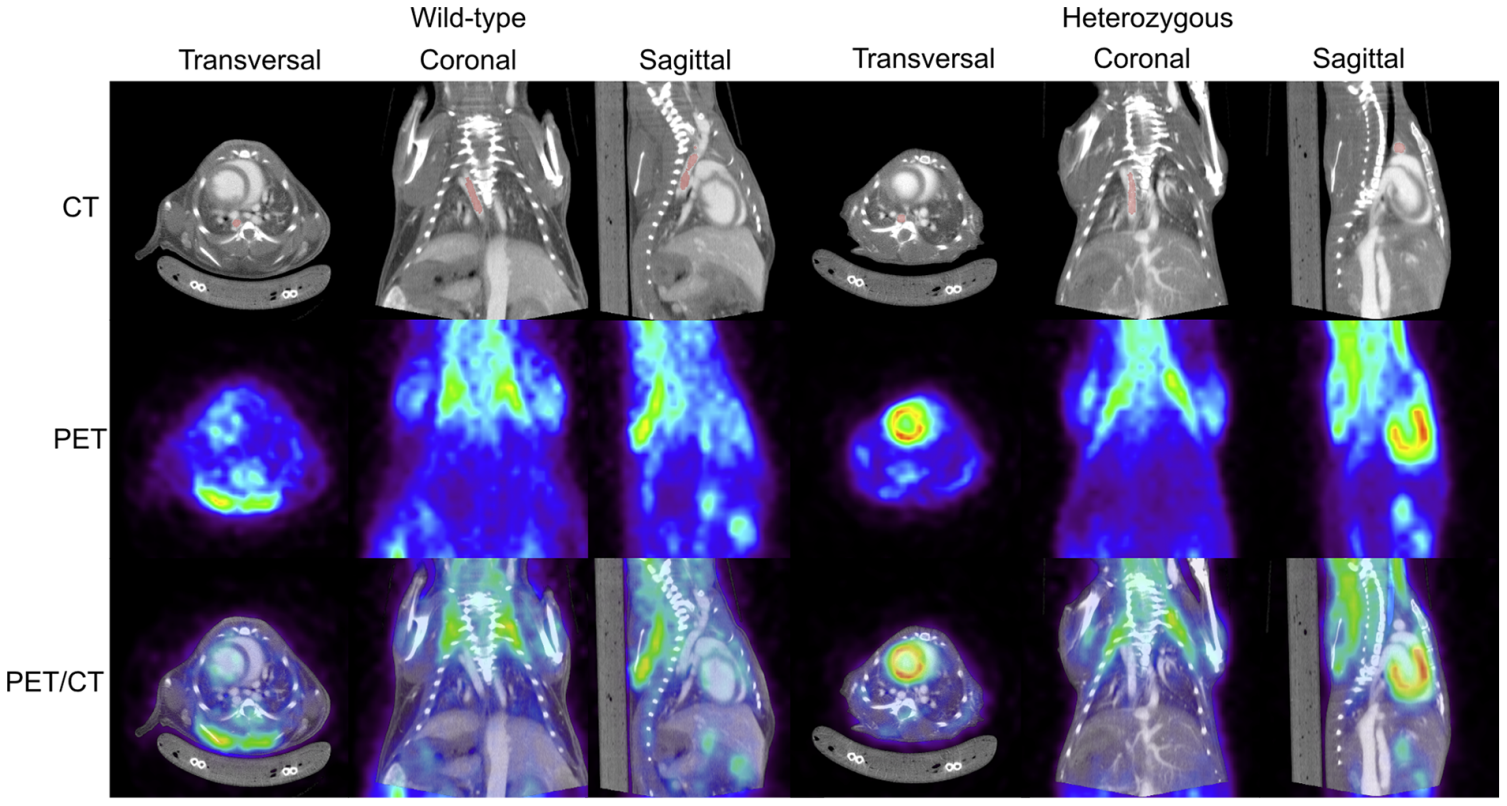
PET imaging of wild-type and heterozygous *Tgfbr1* mice (6 months old). No FDG-PET activity (green-yellow) is seen in heterozygous mice that co-localizes with the thoracic aorta. Top row: Micro-CT reconstruction with thoracic aorta segmentation in red. Middle row: PET. Bottom row: PET/CT.

We studied the cardiovascular system of heterozygous p.Y378* mice and their sex-matched wild-type littermates at 1, 3, 6 and 12 months of age. Experiments were initiated in the groups aged 6 and 12 months. Several invasive and non-invasive imaging techniques were applied for detailed investigation of the cardiovascular system with special attention paid to the aorta. Initial studies with echocardiography of the 6-month-old mice showed no significant differences in aortic diameter, valvular function or left ventricular dimension and function between heterozygous mutant and wild-type mice, as was also the case at 12 months of age (Table S2). Micro-CT was performed at both time points and images were reconstructed and segmented to create a 3 dimensional (3-D) model of the aorta. No differences in aortic diameter were noted between heterozygous p.Y378* and wild-type mice, neither at 6 nor at 12 months of age (Figure 2). Furthermore, no tortuosity of the aorta or branching vessels was observed in the mutant mice. Perturbation of normal elastin laminar structure may arise from induction of TGFβ regulated matrix metalloproteinases (MMP), a family of endopeptidases responsible for the degradation of the extracellular matrix in aortic aneurysms [30]. This increased activity of MMPs can be assessed using *ex vivo* fluorescence reflectance imaging. We compared aortas from *Tgfbr1* mutant mice to wild-type littermate controls and observed no difference in the intensity of the fluorescent signal, excluding minor molecular changes at the level of MMPs in the aortas of *Tgfbr1* muatnt mice (Figure S2). PET-CT was performed to investigate the presence of an inflammatory reaction that may precede aneurysm formation. On the PET images no inflammatory activity was seen that co-localized with the aorta (Figure 3). Also vascular corrosion casting, a technique allowing us to construct a plastic replica of the arterial system of the mice, did not demonstrate any aortic aneurysms or tortuosity of the aorta and/or its major branches, including the carotid arteries, both at 6 and 12 months of age (Figure 2, Table S3). Due to the complete absence of a cardiovascular phenotype in these older mice, it is unlikely that younger mice present any features. Therefore, imaging and further functional analyses of the 1- and 3-month-old mice were no longer scheduled.

### Nonsense mediated mRNA decay

**Figure 4:**
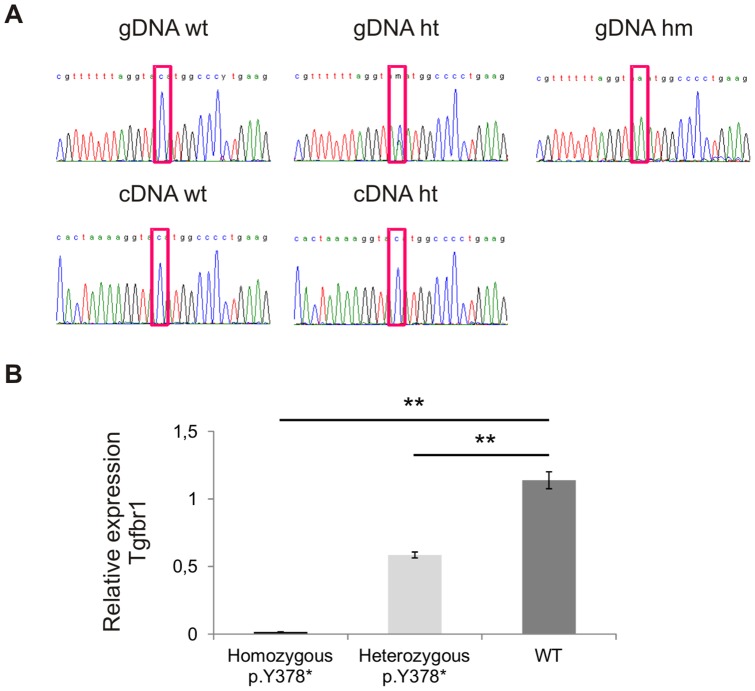
Nonsense mediated mRNA decay of the mutant *Tgfbr1* allele. (**A**) Sequencing of the amplified cDNA confirms the absence of the mutant allele in heterozygous (ht) and homozygous mutant mice (hm) compared to wild-type mice (wt). (**B**) qPCR indicates (near) absence of *Tgfbr1* mRNA in homozygous mutant mice (black bar), and an average of 50% reduction in heterozygous mutant mice (light grey bar) compared to wild-type mice (dark grey bar) (** indicates that p<0.05). Some natural biological variation is seen within the groups (data not shown). Y-axis indicates relative expression of *Tgfbr1* mRNA. gDNA: genomic DNA; cDNA: complementary DNA.

In a next step, the reason for the absence of a disease phenotype in the *Tgfbr1* mouse model was investigated. The nonsense mutation p.Y378* is located at the beginning (second amino acid residue) of exon 7, the last but two coding exon of the *Tgfbr1* gene. Hence, according to the rules of nonsense mediated mRNA decay (NMD), the mutant mRNA is expected to be degraded in this mouse model. In order to detect NMD we first attempted to amplify cDNA from homozygous mutant embryos. When NMD takes place, no mutant cDNA is expected to be present in the homozygous mice, which could be confirmed here (data not shown). Subsequent sequencing of the PCR products revealed the (near) absence of the mutant allele carrying the C to A substitution in the heterozygous mice (Figure 4A). In a second step, the sequencing results were validated using qPCR. These experiments confirmed the ±50% reduction in expression of *Tgfbr1* mRNA in heterozygous embryos and the almost complete absence of *Tgfbr1* mRNA in homozygous mutant embryos (p<0.05) (Figure 4B).

### Aortic wall and TGFβ signaling

**Figure 5:**
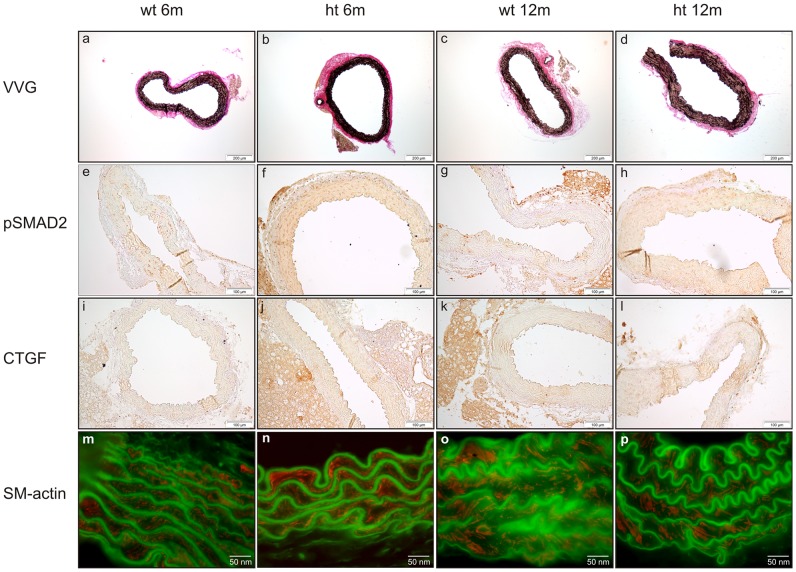
(Immuno)histological and immunofluorescent staining of murine aortic tissue. Upper panel: Verhoeff-Von Gieson (VVG) staining shows intact elastic fibers in both wild-type (wt) (a,c) and heterozygous (ht) *Tgfbr1* mice (b,d) of 6 and 12 months of age. Middle panels: no upregulation of the TGFβ signaling pathway, characterized by increased cytoplasmatic CTGF staining (i–l) and accumulation of nuclear pSmad2 staining (e–h), is seen in heterozygous 6 months (f and j) and 12 months (h and l) old mice compared to wild-type mice (e, i, g and k). Lower panel: aortic tissue of heterozygous *Tgfbr1* mice (n and p) is not characterized by smooth muscle cell loss or focal hyperproliferation of smooth muscle cells compared to wild-type mice (m and o) (red staining). SM-actin: smooth muscle α-actin.

The architecture of the aortic wall in heterozygous p.Y378* mice was similar to that of their wild-type littermates. Fragmentation of elastic fibers, one of the main characteristics of aortic media degeneration, could not be detected upon Verhoeff-Von Giesson elastin staining (Figure 5 a–d). Also, smooth muscle α-actin staining showed no smooth muscle cell loss in heterozygous *Tgfbr1* mutant mice (Figure 5 m–p).

**Figure 6:**
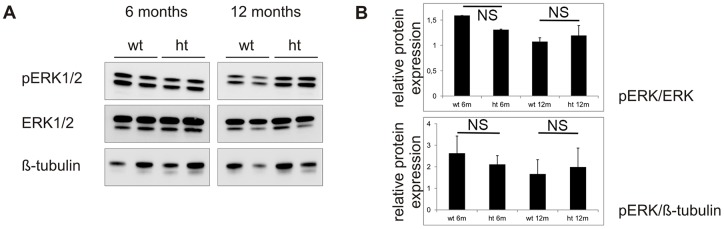
Western blot of non-canonical TGFβ signaling pathway. (**A**) Western blot analysis of pERK1/2 (44 kDa band pERK1 and 42 kDa band pERK2) in wild-type (wt) and heterozygous mutant (ht) mice of 6 and 12 months old shows no major increase or decrease of the active (phosphorylated) form of ERK1/2 in these groups compared to total ERK1/2 and β-tubulin. (**B**) The graphs show normalization of pERK1/2 to total ERK1/2 and β-tubulin protein expression. Y-axis indicates relative protein expression. NS: not significant.

In order to investigate upregulation of the canonical TGFβ signaling pathway as observed in human LDS, we performed immunohistochemical staining of pSmad2 and CTGF on aortic tissue of 6- and 12-month-old mice (Figure 5 e–l). No significant differences in pSmad2 and CTGF staining were observed between heterozygous mutant mice and wild-type mice at both time points. Despite several attempts, our immunohistochemical data could not be verified by western blot. Following recent findings of increased non-canonical TGFβ signaling in an MFS mouse model, we investigated pERK1/2 levels by western blot (Figure 6). No major differences in the amount of active ERK1/2 (pERK1/2) were observed between wild-type and heterozygous mutant mice at both time points (p>0,05).

## Discussion

The principal aim of this study was to develop a mouse model for LDS allowing investigation of the role of TGFβ signaling in aortic aneurysm formation. We also aimed to develop a robust protocol for cardiovascular phenotyping in mice, combining functional, anatomical and molecular imaging with in depth histological characterization of the aortic wall. The *Tgfbr1* nonsense mouse model was selected based on the available data at that time, including observations that nonsense mutations in the *TGFBR2* gene result in LDS in humans, and conditional (neural crest) *Tgfbr1* and *−2* knock-out mice display important cardiovascular abnormalities [21], [22]. During the course of this study, *TGFBR1* nonsense mutations have also been identified in human LDS, further reinforcing the choice for this model.

In our new *Tgfbr1* mouse model, the phenotype was limited to early embryonic lethality of homozygous mutant mice. Heterozygous mice did not display a cardiovascular disease phenotype, which is in line with previous findings in both *Tgfbr1* and *Tgfbr2* knock-out mouse models [19], [20]. The lack of vascular, anatomical or immunohistological anomalies in the heterozygous mutant mice is in contrast with the manifestations encountered in humans harbouring mutations in the TGFβ receptor genes. Both missense and premature termination codon mutations in these genes give rise to LDS in humans ([14], [31], personal communication C. Boileau and E. Arbustini). The effect of the *TGFBR1 and −2* nonsense mutations has not been described yet. However, based on the theoretical rules of the nonsense mediated mRNA decay (NMD) process one could expect the reported *TGFBR2* nonsense mutations to escape this process, because these mutations are all located in the final coding exon or within less than 50–55 nucleotides before the final exon-exon junction and as such the exon junction complexes will be displaced after initial translation and these complexes will not serve as assembly platforms for NMD factors. Possibly, these truncating mutations act in a moderate dominant-negative manner similar to what was observed for missense mutations [16]. In contrast, the nonsense mutations identified in *TGFBR1* are both located before this theoretical ‘boundary’ and as such are predicted to result in NMD. Whether this is indeed the case for both the *TGFBR1* and *−2* nonsense mutations is not known so far. The 2 *TGFBR1* splice site mutations identified by the group of C. Boileau affect 3′ and 5′ splicing of intron 4 (personal communication C. Boileau). Unfortunately, no biological material is available to assess whether these mutations lead to NMD. Although knowledge of the mutation spectrum in LDS increased over the years, the exact underlying disease mechanism remains elusive. However, the existence of a haploinsufficient disease mechanism seems unlikely, since heterozygous *Tgfbr1* and *−2* knock-out mice develop normally, and have a normal life span [19], [20]. Human data in support for the absence of a haploinsufficiency model include a study published by Redon and colleagues, who identified a heterozygous *de novo* microdeletion (9q22.32-q22.33), encompassing the *TGFBR1* gene, in two unrelated patients who present overgrowth and psychomotor delay but do not show any of the typical LDS features [32]. Another argument against a haploinsufficiency disease mechanism is the demonstration of a mild dominant-negative effect of *TGFBR1* and *TGFBR2* missense mutations in human HEK293 cell cultures [15], [16]. For instance, co-transfection of mutant *TGFBR1* reporter constructs with an eGFP (enhanced green fluorescent protein)-tagged Smad2 construct showed that missense mutations in the serine/threonine kinase domain of the type I receptor either affected the activation of TGFβ receptor type I by the type II receptor, or inhibited the phosphorylation of receptor-Smads [15]. Co-transfection of equal amounts of wild-type and mutant *TGFBR1* revealed a modest downregulation of TGFβ signaling, providing evidence for, at least a moderate, dominant-negative effect of the mutant receptor [15]. Here, we show that our model does not recapitulate the LDS phenotype and therefore our study further confirms that a *TGFBR1* haploinsufficient disease mechanism is very unlikely in LDS, as the development of the cardiovascular system remains unaffected notwithstanding the 50% reduction in *Tgfbr1* mRNA in heterozygous animals. Nonetheless, haploinsufficiency of other TGFβ signaling components, such as the SMAD3 effector and the TGFβ2 ligand, which also leads to pathologically increased TGFβ signaling has previously been suggested as the disease mechanism causing thoracic aortic aneurysms and various associated features [24], [33], [34].

Recently, *TGFBR1* nonsense mutations have been identified in patients with multiple self-healing squamous epithelioma (MSSE, MIM 132800) [37]. As opposed to LDS, in which predominantly missense mutations in the kinase domain of the receptor lead to a cardiovascular, neurocognitive and craniofacial-skeletal phenotype, only truncating mutations are identified in the kinase domain of the receptor in MSSE patients, together with truncating and missense mutations in the ligand-binding extracellular domain of the type I receptor. As such, the differences in the spectrum and location of mutation types determine the phenotype. A macroscopic evaluation of the skin of fifteen 12-month-old heterozygous *Tgfbr1* mice did not reveal any scars as observed in human MSSE patients after spontaneous regression of the skin tumors. More thorough assessment of the skin of this model, for example by challenging it with known cancerogens, is needed to evaluate the skin phenotype.

Other possible explanations for the absence of a disease phenotype in these mice should also be considered. First, the decrease in Tgfbr1 signaling that ought to be caused by haploinsufficiency could be compensated for by other receptors of the TGFβ superfamily. For instance, it was shown by Carvalho and co-workers that the ALK4 receptor is able to phosphorylate SMAD2 in a conditional *Tgfbr1^−/−^* (also known as *Alk5*) knock-out mouse model, compensating for the reduction in Tgfbr1 signaling [17]. Second, reduced penetrance of the phenotype might relate to the genetic background (black 6) of the mouse strain used in this study. This hypothesis is supported by the study of Tang and colleagues, who identified three unlinked genetic modifiers which may regulate TGFβ activation as shown by the survival-to-birth rate in different strains in which *Tgfb1* was deleted [35], [36].

Since the TGFβ signaling pathway is thought to play a crucial role in the pathophysiology of thoracic aortic aneurysms and the therapeutic implications hereof, it is of utmost importance to unravel the precise role of the TGFβ signaling pathway in aortic disease and understand the seemingly paradoxical upregulation of this pathway in LDS patients. Mouse models bearing missense mutations previously identified in human LDS patients could possibly model the disease and be instrumental in identifying it's pathogenetic mechanism.

In conclusion, the haploinsufficient *Tgfbr1* mouse model created in this study does not recapitulate the human LDS phenotype. Consequently, we were not able to elucidate the TGFβ paradox that has been previously proposed to occur in LDS. In view of the similarity to mutations identified in MSSE, this model might be valuable for further studies of the pathogenesis of this phenotype. Furthermore, with the combination of detailed phenotyping techniques, we provide a useful protocol for the characterization of cardiovascular features in mice. Our protocol may be of practical use for other researchers in the field.

## Supporting Information

Figure S1 **Sites of measurement of the thoracic aorta diameters on echocardiographic evaluation.** (1) aortic annulus, (2) sinus aortae, (3) sinotubular junction, (4) ascending aorta, (5) aortic arch, and (6) descending aorta.
(TIF)

Figure S2 **Fluorescence imaging of MMP activity in aortas from wild-type and heterozygous** ***Tgfbr1*** **mice.** Near infrared fluorescence images showing the fluorescence of MMPsense 680 *ex vivo* in the thoracic aorta at 24 hours post-injection. Shown are an uninjected control aorta, 2 wild-type and 2 heterozygous *Tgfbr1* aortas.
(TIF)

Table S1 **Overview of reported and unpublished** ***TGFBR1*** **and** ***TGFBR2*** **premature termination codon mutations identified in LDS patients.** CMGG: Center for Medical Genetics Ghent.
(XLSX)

Table S2 **Echocardiographic data of wild-type and mutant** ***Tgfbr1*** **mice at 6 and 12 months of age.** WT: wild-type; Stdev: standard deviation; LVEDD: left ventricular end diastolic diameter; LVESD: left ventricular end systolic diameter. FS: fractional shortening; E: early transmitral flow velocity; A: late transmitral flow velocity; DT: deceleration time of E velocity, E/A: ratio of early (E) to late (A) peak velocities; Em: early mitral annulus velocity; E/E': relationship between maximal values of passive mitral inflow.
(XLSX)

Table S3 **Aortic diameters measured from vascular corrosion casts of wild-type and mutant** ***Tgfbr1*** **mice at 6 and 12 months of age.** WT: wild-type; Stdev: standard deviation.
(XLSX)
