# Supplementary figures and images for: Absence of Cardiovascular Manifestations in a Haploinsufficient Tgfbr1 Mouse Model
Source: PLoS One. 2014 Feb 24;9(2):e89749. doi: 10.1371/journal.pone.0089749 (PMC3933654; doi:10.1371/journal.pone.0089749)

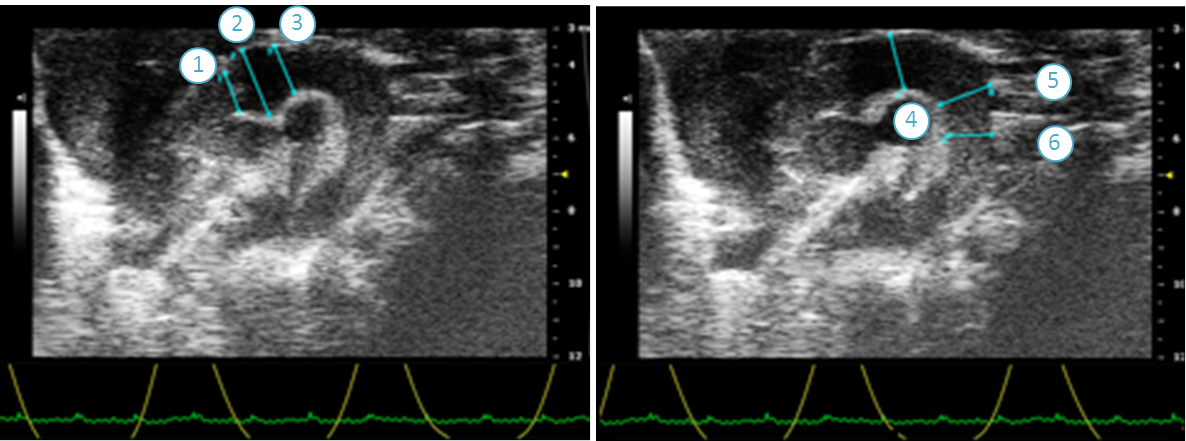

Supplement: Figure S1 — Sites of measurement of the thoracic aorta diameters on echocardiographic evaluation. (1) aortic annulus, (2) sinus aortae, (3) sinotubular junction, (4) ascending aorta, (5) aortic arch, and (6) descending aorta. (TIF) [file pone.0089749.s001.tif]

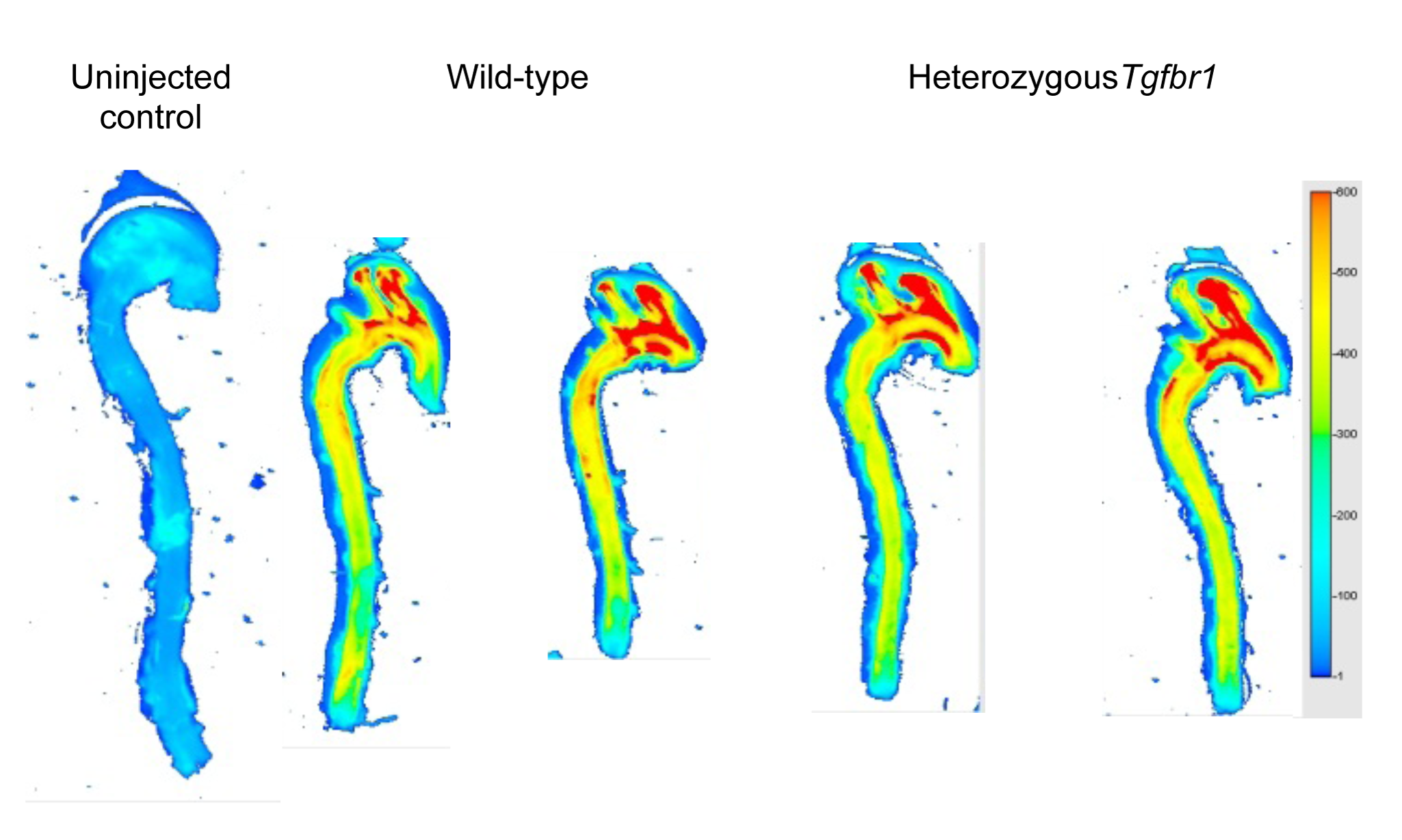

Supplement: Figure S2 — Fluorescence imaging of MMP activity in aortas from wild-type and heterozygous Tgfbr1 mice. Near infrared fluorescence images showing the fluorescence of MMPsense 680 ex vivo in the thoracic aorta at 24 hours post-injection. Shown are an uninjected control aorta, 2 wild-type and 2 heterozygous Tgfbr1 aortas. (TIF) [file pone.0089749.s002.tif]
